# Brief Educational Workshops in Secondary Schools Trial (BESST): a cluster randomised controlled trial. Secondary analysis in those with elevated symptoms of depression

**DOI:** 10.1136/bmjment-2024-301192

**Published:** 2024-08-29

**Authors:** Stephen Lisk, Kirsty James, James Shearer, Sarah Byford, Paul Stallard, Jessica Deighton, David Saunders, Jynna Yarrum, Peter Fonagy, Timothy Weaver, Irene Sclare, Crispin Day, Claire Evans, Ben Carter, June Brown

**Affiliations:** 1 Department of Psychology, Institute of Psychiatry, Psychology and Neuroscience, King's College London, London, UK; 2 King’s Clinical Trials Unit, Institute of Psychiatry, Psychology and Neuroscience, King's College London, London, UK; 3 Department of Biostatistics and Health informatics, Institute of Psychiatry, Psychology and Neuroscience, King's College London, London, UK; 4 Health Service and Population Research Department, Institute of Psychiatry, Psychology and Neuroscience, King's College London, London, UK; 5 Department for Health, University of Bath, Bath, UK; 6 Anna Freud National Centre for Children and Families, London, UK; 7 Faculty of Health, Education and Society, University of Northampton, Northampton, UK; 8 Department of Clinical, Educational and Health Psychology, University College London, London, UK; 9 Department of Mental Health and Social Work, Middlesex University, London, UK; 10 Southwark CAMHS Clinical Academic Group, South London and Maudsley Mental Health NHS Trust, London, UK

**Keywords:** Depression & mood disorders, Child & adolescent psychiatry, Anxiety disorders, Depression

## Abstract

**Background:**

Depression and anxiety are increasingly prevalent in adolescents. The Brief Educational Workshops in Secondary Schools Trial investigated the effectiveness of a brief self-referral stress workshop programme for sixth-form students aged 16–18 years old.

**Objective:**

This study conducted a secondary analysis on the outcomes of participants with elevated depressive symptoms at baseline.

**Methods:**

This is an England-wide, multicentre, cluster randomised controlled trial to evaluate the clinical effectiveness and cost-effectiveness of a brief cognitive–behavioural therapy workshop (DISCOVER) compared with treatment-as-usual (TAU) (1:1). The primary outcome was depression symptoms (Mood and Feelings Questionnaire (MFQ)) at 6-month follow-up, using the intention-to-treat (ITT) population and analysed with a multilevel linear regression estimating a between-group adjusted mean difference (aMD). Cost-effectiveness, taking a National Health Service (NHS) and personal social services perspective, was explored using quality-adjusted life years (QALYs).

**Findings:**

Between 4 October 2021 and 10 November 2022, 900 adolescents at 57 schools were enrolled. 314 students were identified as having elevated symptoms of depression at baseline (>27 on MFQ). In this prespecified subgroup, the DISCOVER arm included 142 participants and TAU included 172. ITT analysis included 298 participants. Primary analysis at 6 months found aMD to be −3.88 (95% CI −6.48, –1.29; Cohen’s d=−0.52; p=0.003), with a similar reduction at 3 months (aMD=−4.00; 95% CI −6.58, –1.42; Cohen’s d=0.53; p=0.002), indicating a moderate, clinically meaningful effect in the DISCOVER arm. We found an incremental cost-effectiveness ratio of £5255 per QALY, with a probability of DISCOVER being cost-effective at between 89% and 95% compared with TAU.

**Conclusions and clinical implications:**

DISCOVER is clinically effective and cost-effective in those with elevated depressive symptoms. This intervention could be used as an early school-based intervention by the NHS.

**Trial registration number:**

ISRCTN90912799.

**WHAT IS ALREADY KNOWN ON THIS TOPIC**

**WHAT THIS STUDY ADDS**

- This study used a self-referral approach alongside factors identified as necessary for effective school-based interventions (cognitive–behavioural therapy-focused, secondary school-based, delivered by clinicians) to provide new evidence on the effectiveness of a school-based stress workshop delivered by MHSTs.
- This self-referral workshop programme for students aged 16–18 years old has demonstrated it can reach and assist participants with elevated depressive symptoms, both clinically and cost-effectively.

**HOW THIS STUDY MIGHT AFFECT RESEARCH, PRACTICE OR POLICY**

- This intervention could be used by the National Health Service as an early intervention in schools.

## Background

 Emotional disorders such as anxiety and depression are particularly common in adolescence, causing significant distress and disruption in about 1 in 12 (8.1%) young people in England.^1^ Most childhood and adolescent anxiety disorders go untreated, with less than a quarter of anxious and depressed youth aged 12–17 having contact with specialist child and adolescent mental health services (CAMHS) in the UK. This is often due to concerns about stigma and confidentiality,^2^ as well as limited capacity and stringent eligibility criteria for specialist mental health services. There is a pressing need for accessible and evidence-based interventions for students at risk of depression.

### Treatment access

Educational settings are uniquely situated to offer the first level of accessible mental health support and move away from the constraints of secondary care referral systems.^3^ In the UK, government policy has increasingly focused on schools/colleges to assist in delivering adolescent interventions.^4^ Mental health support teams (MHSTs) have been established to facilitate targeted mental health support within schools.

However, there are mixed results on the success of psychological interventions delivered in school settings. Systematic reviews have concluded that while school-based interventions generally show a weak to moderate effect in reducing anxiety, there have been no consistent results regarding the effectiveness in reducing depression.^5 6^ Cognitive–behavioural therapy (CBT) is a widely used approach to treating depressive symptoms, with a significant evidence base.^7^ Research also indicates that a range of social–emotional learning curricula, when implemented with care, can result in positive outcomes.^8 9^ Additionally, some recent studies have raised questions about the universal application of school-based CBT approaches, suggesting that their effectiveness may vary, and in some cases unintended negative effects may occur.^10^

However, a recent meta-analysis of randomised controlled trials (RCTs) for school-aged children and adolescents identified key factors for successful school-based interventions: interventions using CBT, delivery in secondary school settings and interventions led by clinicians.^5^

### Targeted approach

Most existing school-based interventions focus on either targeted or universal approaches. Targeted interventions are designed to only include students who have mental health issues, usually through screening or diagnostic assessment. These have generally demonstrated greater effectiveness than universal programmes, especially in secondary schools.^11^ However, targeted interventions can leave students feeling stigmatised due to the visibility of the screening process.^12^ Moreover, this approach may miss students who do not exhibit overt symptoms or who are reluctant to seek help due to fear of negative social evaluation.^2^

### Universal approach

Universal mental health interventions aim to address these issues by offering preventive programmes to all students. These interventions are increasingly popular,^5^ with teachers often trained to deliver the programmes instead of clinicians. However, universal approaches are less effective in reducing mental health issues for those who already exhibit problems.^13 14^ Furthermore, less favourable outcomes have been reported when teachers lead the programmes compared with clinicians.^13 15^ Additionally, assessing the effectiveness of universal programmes can be problematic as average scores may obscure the impact of interventions on specific subgroups, such as those students who are more susceptible to mental health issues. A recent universal mindfulness trial conducted in secondary schools reported no significant changes in depression overall, but an increase in depressive symptoms following the intervention for adolescents with elevated mental health issues at baseline compared with the control group.^13^

### Self-referral approach

A self-referral approach to recruitment may address these challenges and provide other advantages. This strategy places the decision-making power with the participants themselves, empowering them to take control of their mental health by allowing them to self-identify their needs and enrol for support being offered. It also aims to increase access and reduce stigma by eliminating ‘screening’ processes. Brown and colleagues^16^ developed the community self-referral approach with CBT stress workshops for adults and found that 39% of participants had not previously sought help through their General Practitioner (GP). Self-referral has also been shown to engage over 70% of adults with diagnosable mental health problems in self-referral stress workshops.^17^

This self-referral workshop approach has been adapted for adolescents aged 16–18 years old: the DISCOVER workshop,^18^ delivered in school sixth forms. A feasibility study showed that the workshops reached 66% of students who had not previously sought support. The workshops were inclusive, with 55% of participants coming from ethnic minority groups, and although underpowered the workshops demonstrated a postworkshop reduction in depressive symptoms.^19^

### Objective of this study

This paper reports a secondary analysis of data from a previously registered trial (ISRCTN90912799). The Brief Educational Workshops in Secondary Schools Trial (BESST) aimed to rigorously evaluate the effectiveness of this self-referral workshop through a cluster RCT. Whole-group analyses have already shown the intervention to be more effective than usual school provision in reducing symptoms of depression and anxiety and improving well-being and resilience at 6 months.^20^ This study aims to test how well this self-referral workshop programme impacted those students exhibiting elevated depressive symptoms.

Specifically, we aim to address the clinical effectiveness of the intervention in depression among students exhibiting elevated depressive symptoms, the effect of the intervention on secondary outcome measures (anxiety, well-being, resilience, sleep) in this group and the cost-effectiveness of the intervention for this group.

## Methods

### Study design

**Figure 1:**
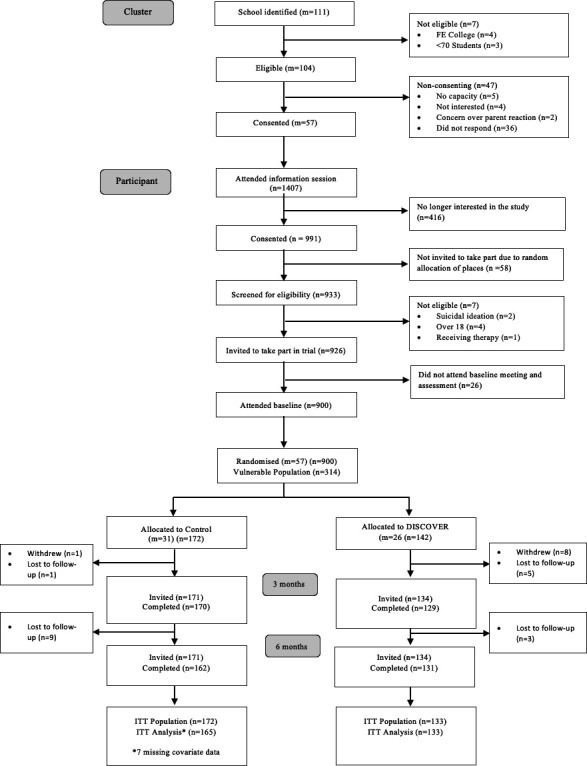
CONSORT diagram. CONSORT, Consolidated Standards of Reporting Trials; FE, Further Education; ITT, intention-to-treat; n, number of individuals; m, number of clusters.

In this study, we conducted secondary analyses using data from BESST (figure 1). BESST is a two-arm cluster RCT^21^ comparing a psychological stress workshop (ie, DISCOVER) versus normal school provision (treatment-as-usual (TAU)). The DISCOVER and TAU programmes are described in the online supplemental materials. We recruited and randomised 900 adolescents (age 16–18 years at baseline) in 57 schools across England. Of the 900 participants who were recruited into the study, 314 (35%) scored above the recommended cut-off on the baseline Mood and Feelings Questionnaire (MFQ) (>27).^20^ We will refer to this as the ‘elevated depressive symptoms’ group. Consistent with the protocol, we used data from baseline and 3-month and 6-month follow-ups. Study design and procedures are presented in full in the study protocol^21^ and main trial outcomes paper.^20^

### Participants

Recruitment was conducted in two cohorts (recruited in academic years 2021–2022 and 2022–2023) and involved consenting schools, then seeking consent directly from young people. Participants were aged 16–18 years, attending for the full school year, seeking psychological help for stress, fluent in English and able to provide informed written consent. Participants were excluded if they were identified as actively suicidal, had severe learning difficulties and were actively receiving psychological therapy for anxiety or depression through CAMHS.

### Intervention

DISCOVER is a brief, accessible workshop-based stress management programme for (and codesigned by) adolescents aged 16–18 years old, to which they can self-refer. Delivered by one senior and two junior therapists, the workshop programme consists of (1) a *preworkshop 1-2-1 meeting* between a workshop leader and each student; (2) a *1-day group workshop* using CBT-informed model of emotional problems to explain and normalise young people’s experiences and provide techniques and strategies for managing anxiety and mood issues; and (3) *postworkshop individual telephone support* from the workshop leaders to monitor goal attainment progress and assistance in applying CBT skills in real-life situations. Self-report and observer fidelity was measured. See online supplemental appendix S1 for a full description of the intervention and fidelity.

### Procedures

The study implemented a self-referral approach when recruiting students into the trial. This aligns with the approach used by the DISCOVER workshop programme in real-world settings, where the programme is promoted to students using non-stigmatising language, emphasising the students’ autonomy in deciding whether to participate without the need for screening measures.

In this study, all sixth-form students were introduced to the trial during an assembly presentation by a trial researcher. This presentation employed the above strategy to highlight what the trial (and workshop) entailed and who it was most suitable for, while avoiding stigmatising language or labelling of those considering enrolment. It was made clear there would be no form of diagnostic screening to select participants; it was ensured that the students understood they were trusted to decide whether this was something they felt would be suitable for them. Any interested students were invited to a meeting in school where they were given more information about the study and provided with a written information sheet and consent form, along with an opportunity to ask questions.

Once consented, a baseline assessment was conducted in a private room at the school, where a blinded research worker reviewed the inclusion/exclusion criteria with each participant. The participants then completed baseline questionnaires. It was explained to each participant that these confidential assessments were for research purposes only. Following the baseline timepoint, participants in sixth forms allocated to the TAU arm received usual school care as well as a signposting information sheet that was provided to all trial participants. Participants in sixth forms allocated to the DISCOVER arm were invited to attend the workshop programme delivered at their sixth form.

### Outcomes

The outcomes used are consistent with the primary analysis for BESST.^21^ Depression was assessed using the MFQ.^22^ Anxiety was assessed using the anxiety subscale of the Revised Child Anxiety and Depression Scale-Child Version.^23^ Well-being was assessed using the Warwick-Edinburgh Mental Well-being Scale.^24^ Sleep quality was assessed using the Sleep Condition Indicator.^25^ Resilience was assessed using the Child and Youth Resilience Measure-12.^26^ Student satisfaction measured in the intervention arm only was assessed using the Client Satisfaction Questionnaire.^27^ Health-related quality of life was assessed using the EuroQol 5-Dimension, 3-Level(EQ-5D-3L) questionnaire.^28^ Use of health and social care services was assessed using the Child and Adolescent Service Use Schedule (CA-SUS).

### Statistical analysis

The primary and secondary outcome analyses from BESST were repeated for the elevated depressive symptom group only. All outcomes were analysed using a mixed-effect, multilevel linear model at 6 months, adjusted using fixed effects of baseline severity of the respective outcome, aggregated level school deprivation, geographical area, school size, gender, ethnic group, time, treatment and treatment-by-time interaction. A random intercept was fitted for each school and student, and the difference between the intervention and the control score was estimated, alongside 95% CI and p value. All participants with ‘elevated depressive symptoms’ with follow-up data were included in the ‘elevated depressive symptoms’ intention-to-treat (ITT) population. To mitigate type I errors, we followed a prepublished protocol and statistical analysis plan with power calculations.

### Health economic analysis

The economic analyses from BESST were repeated for the elevated depressive symptom group only. The primary economic analysis was a cost-utility analysis at 6 months, with quality-adjusted life years (QALYs) calculated using the EQ-5D-3L measure of health-related quality of life as the measure of effectiveness.^28^ Health and social care costs, including the cost of the BESST intervention, were analysed from the National Health Service (NHS)/personal social services perspective preferred by the National Institute for Health and Care Excellence (NICE).^29^ The BESST workshop intervention, including training and supervision of facilitators, workshop delivery and follow-up phone calls, was costed using a micro-costing approach and is detailed in full in the main outcome paper. Nationally applicable unit costs were applied to other health and social service use collected using the CA-SUS.

Costs and QALYs adjusted for baseline covariates, including aggregated level school deprivation, geographical area, school size, gender and BME group, were compared between groups using generalised linear modelling with appropriate family and link functions and bootstrapped CIs as recommended to account for the highly skewed nature of cost data.^30^ Cost-effectiveness was explored in terms of cost per QALY using incremental cost-effectiveness ratio (ICER), with uncertainty represented by cost-effectiveness acceptability curves, which show the probability that an intervention is cost-effective compared with control across a range of willingness-to-pay thresholds.^31^

## Findings

We randomised 57 sixth forms, which included 314 students with elevated symptoms of depression.

The baseline characteristics of the population (N=314) are presented in table 1. Participants were predominantly female (241, 76.8%) and just under half (148, 47.2%) were from ethnic minority groups, consistent with the percentage split for the entire sample of 900 students in the main study.^20^ In this group with elevated depressive symptoms, only 29.3% had previously sought help from their GP for mental health problems.

**Table 1:** Baseline characteristics of the intention-to-treat (ITT) population

Baseline characteristics	Control (n=172) n (%)	DISCOVER (n=142) n (%)	Overall (N=314) n (%)
Age, mean (SD)	17.2 (0.6)	17.3 (0.6)	17.2 (0.6)
Gender			
Male	25 (14.5)	29 (20.4)	54 (17.2)
Female	134 (77.9)	107 (75.4)	241 (76.8)
Other	13 (7.6)	6 (4.2)	19 (6.1)
Ethnicity			
White	86 (50.0)	72 (50.7)	158 (50.3)
Mixed	12 (7.0)	10 (7.0)	22 (7.0)
Asian	25 (14.5)	28 (19.7)	53 (16.9)
Black	31 (18.0)	22 (15.5)	53 (16.9)
Other/missing	18 (10.5)	10 (7.0)	28 (8.9)
Sixth form/college year			
Year 1	88 (51.2)	62 (43.7)	150 (47.8)
Year 2	84 (48.8)	80 (56.3)	164 (52.2)
English as the first language			
No	19 (11.0)	19 (13.4)	38 (12.1)
Yes	153 (89.0)	123 (86.6)	276 (87.9)
Number of GCSEs passed, mean (SD)	8.4 (1.8)	8.4 (1.7)	8.4 (1.7)
Participant IMD, mean (SD)	4.2 (2.7)	4.6 (2.9)	4.4 (2.8)
Previously sought help from GP for mental health			
No	121 (70.3)	101 (71.1)	222 (70.7)
Yes	51 (29.7)	41 (28.9)	92 (29.3)

GCSE, General Certificate of Secondary Education; GP, General Practitioner; IMD, Index of Multiple Deprevation.

### Clinical effectiveness

**Figure 2:**
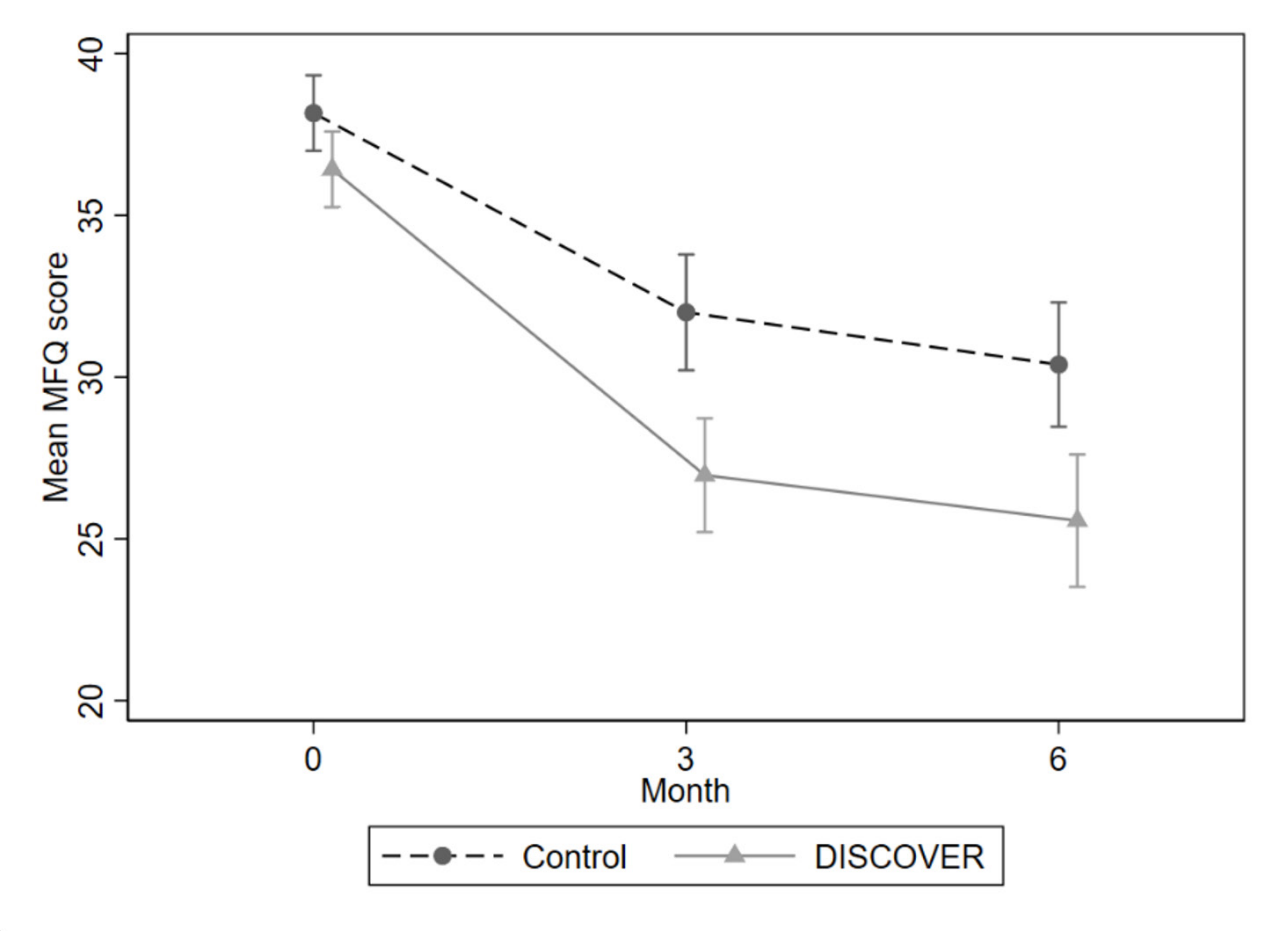
Mean MFQ temporal plot. MFQ, Mood and Feelings Questionnaire.

All raw outcome data are presented in table 2. The ITT analysis included 298 participants. At 6 months, we found an adjusted mean difference (aMD) of −3.88 (95% CI −6.48, –1.29; Cohen’s d=−0.52 (95% CI –0.86, –0.17), p=0.003), showing a clinically meaningful statistically significant greater reduction in depressive symptoms in the DISCOVER arm versus control (figure 2).

**Table 2:** Raw outcome data

Outcome measure	Timepoint								
	Baseline			3 months			6 months		
	Control (n=172) Mean (SD)	DISCOVER (n=142) Mean (SD)	Overall (N=314) Mean (SD)	Control (n=171) Mean (SD)	DISCOVER (n=134) Mean (SD)	Overall (n=305) Mean (SD)	Control (n=171) Mean (SD)	DISCOVER (n=134) Mean (SD)	Overall (n=305) Mean (SD)
Primary outcome									
MFQ									
n	172	142	314	169	129	298	162	131	293
Total score	38.2 (7.8)	36.4 (7.1)	37.4 (7.5)	32.1 (11.9)	27.0 (10.2)	29.9 (11.4)	30.6 (12.5)	25.6 (11.9)	28.3 (12.4)
Secondary outcomes									
RCADS anxiety t-score									
n	157	136	293	158	124	282	151	126	277
Total score	65.8 (10.9)	66.5 (11.1)	66.2 (11.0)	61.9 (12.5)	58.4 (12.1)	60.4 (12.4)	59.5 (13.3)	56.3 (12.7)	58.1 (13.1)
WEMWBS									
n	170	141	311	170	129	299	162	131	293
Total score	33.5 (7.1)	35.5 (7.1)	34.4 (7.2)	36.2 (8.6)	39.5 (9.5)	37.7 (9.2)	37.5 (8.7)	41.6 (8.9)	39.3 (9.0)
Sleep condition indicator									
n	171	142	313	169	129	298	161	129	290
Total score	15.6 (6.3)	15.8 (6.9)	15.7 (6.6)	16.1 (7.4)	17.4 (6.3)	16.7 (7.0)	16.7 (7.7)	18.2 (6.6)	17.4 (7.3)
CYRM-12									
n	172	142	314	169	129	298	161	130	291
Total score	39.8 (7.9)	40.8 (7.1)	40.3 (7.5)	40.0 (7.6)	41.5 (8.4)	40.6 (8.0)	40.4 (7.8)	42.9 (7.7)	41.5 (7.8)

CYRM-12, Child and Youth Resilience Measure-12; MFQ, Mood and Feelings Questionnaire; RCADS, Revised Child Anxiety and Depression Scale; WEMWBS, Warwick-Edinburgh Mental Well-being Scale.

For all other outcomes (table 3), we found a statistically significant improvement in the DISCOVER arm versus control for well-being (Cohen’s d=0.48; 95% CI 0.21, 0.74; p=0.00039), anxiety (Cohen’s d=−0.35; 95% CI −0.57, −0.12; p=0.0023), resilience (Cohen’s d=0.27; 95% CI 0.08, 0.45; p=0.0051) and sleep (Cohen’s d=0.23; 95% CI 0.03, 0.43; p=0.027) (online supplemental figures S1–S5).

**Table 3:** Standardised effect estimates for the primary and secondary outcomes

Outcome		Adjusted mean difference		Cohen’s d		
	n	Estimate	95% CI	Effect	95% CI	P value
Primary outcome						
MFQ	298	-3.88	−6.48, −1.29	−0.52	−0.86, −0.17	0.0033
Secondary outcomes						
WEMWBS	296	3.44	1.54, 5.34	0.48	0.21, 0.74	0.00039
SCI	296	1.51	0.18, 2.85	0.23	0.03, 0.43	0.027
RCADS anxiety t-score	279	-3.81	−6.25, −1.36	−0.35	−0.57, −0.12	0.0023
CYRM-12	298	2.00	0.60, 3.40	0.27	0.08, 0.45	0.0051

CYRM-12, Child and Youth Resilience Measure-12; MFQ, Mood and Feelings Questionnaire; RCADS, Revised Child Anxiety and Depression Scale; SCI, Sleep Condition Indicator; WEMWBS, Warwick-Edinburgh Mental Well-being Scale.

DISCOVER workshop attendance was good, with 88.7% (126 out of 142) attending 75% or more of the workshop (online supplemental table S1). Participant satisfaction with the intervention was high, with a mean score on the Client Satisfaction Questionnaire(CSQ-8) of 26.1 (4.0) for the 116 participants who provided data (online supplemental table S3).

There were 12 adverse events reported overall (TAU=5, DISCOVER=7), with 2 of these events deemed serious (online supplemental table S4). Only one was considered possibly related to study procedures.

### Cost-effectiveness

The cost of the DISCOVER intervention was estimated to be £110.08 per student. Total disaggregated costs per participant over the 6-month follow-up for the elevated depressive symptoms population are reported in online supplemental table S5. The observed costs were higher in the DISCOVER group (mean £1205.57) compared with the TAU group (mean £893.16), and this difference was primarily due to the additional cost of the intervention and higher community service costs. When adjusted for baseline covariates, the total costs per participant remained higher in the DISCOVER group compared with TAU (aMD £113.29, SE £211.16 (95% CI −£739.51, £683.36), p=0.623). The EQ-5D-3L scores and QALYs for the elevated depressive symptoms group are reported in online supplemental table S6. QALYs over the 6-month follow-up were significantly higher in the DISCOVER group than in the TAU group (aMD 0.0226, SE 0.0084 (95% CI 0.0001, 0.0499), p=0.008).

The point estimate of the ratio of the mean difference in costs and QALYs for DISCOVER compared with TAU, referred to as the ICER, was £5314 per QALY, with additional effects generated by DISCOVER being associated with additional costs, as illustrated in online supplemental figure S6. The cost-effectiveness acceptability curve (online supplemental figure S7) shows the probability that DISCOVER is cost-effective compared with TAU ranged from 90.5% to 94.8% at the £20 000–£30 000 per QALY threshold preferred by NICE.^29^

## Discussion

BESST enrolled 900 adolescents in a school-based intervention for older adolescents, 314 of whom were identified as having elevated depressive symptoms (defined as symptoms at baseline >27). This trial demonstrated a clear effect in reducing the depressive symptoms of students with elevated depressive scores, with a moderate effect size at 6-month follow-up. The results also indicated improvements in well-being, anxiety, resilience and sleep.

The reduction in depression scores was closely mirrored by significant improvement in health-related quality of life, which in turn provided substantial evidence for the cost-effectiveness of DISCOVER compared with TAU in young people with elevated depressive symptoms, using the thresholds for value for money commonly used to commission services in the UK.

The success of this intervention for this group of students is notable, especially when considering the lack of success observed in reviews of other school-based interventions in reducing depression.^5^ Additionally, a recent large-scale trial of a school-based mindfulness training programme led by teachers resulted in a deterioration in students vulnerable to depression compared with the control group.^32^ A previous UK study of classroom-based CBT with students aged 11–15 years old (also implementing a universal access approach) found no difference in improvement in depression between the intervention and the TAU group.^10^

The brief CBT stress workshop evaluated here was developed and conducted in secondary schools and led by clinicians. The DISCOVER workshop is based on CBT principles and was co-designed with adolescents aged 16–18 years old to incorporate age-appropriate topics, materials and approaches tailored to the specific needs of this age group. Consistent with previous research, the effect we observed might have been influenced by some of these factors, which have been identified as crucial for successful outcomes in school-based interventions.^5^

It is noteworthy that DISCOVER was effectively delivered with the assistance of a newly introduced professional group of clinicians (MHSTs) who are based in schools and had received training in more low-intensity treatments for mild to moderate problems.^4^ Workshops were delivered by one senior therapist and two more junior therapists from the MHSTs. The positive outcome results demonstrate that, with adequate additional training, MHST staff were capable of effectively delivering the workshops and significantly impacting those students experiencing elevated depressive symptoms.

A distinctive feature of the DISCOVER intervention lies in its accessibility. The self-referral system for recruitment, which was outlined and introduced at the assembly meeting, is very much driven by participants’ own decision-making and aligns with adolescents’ desire for autonomy.^33^ This contrasts with universal or targeted interventions where enrolment is researcher/clinician-led. The high proportion of those who had not previously sought help through formal routes (70%) underscores the value of this approach. Adult studies have also found similar benefits of greater accessibility and increased uptake when offered a self-referral option to mental health support.^34 35^

There are significant advantages to the innovative self-referral approach. It has the potential to reach vulnerable individuals who may otherwise be deterred and feel stigmatised^12^ by the visibility of the screening process in targeted interventions. The self-referral approach also seems to result in the enrolment of a relatively large number of participants from diverse ethnic groups (46%) who require help with these elevated symptoms. It has been highlighted that studies include relatively few participants from ethnic minority groups,^36^ resulting in research studies often showing bias towards white middle-class participants. Complex culturally adapted interventions may sometimes be necessary for different groups; however, this study shows that improving access for younger participants from diverse ethnic groups may be an alternative and potentially effective way of enhancing access. There is increasing interest in self-referral; a recent review found that health inequalities can be reduced with a self-referral system but needs to be targeted carefully.^37^ This is relevant to the issue of accessibility, which is important to the government’s NHS Long Term Plan.

This non-stigmatising self-referral approach, combined with the transdiagnostic and practical nature of the workshop programme, may be of great benefit to students. This approach, which aims to provide participants with greater feelings of autonomy, may also increase their motivation to engage with the intervention and thereby boost the treatment effect. It is also possible that individuals with elevated symptoms may view universal approaches as being ‘watered down’, or not serious enough for their issues, due to the fact they are provided to all students and thereby negatively impact their motivation to engage with the intervention.

However, while we have seen impressive levels of engagement, this approach may not be suitable for some individuals; not all students who came to the information meeting signed up for the study. We need to understand which students this approach is suitable for and which students require a different approach. Additionally, there was a gender imbalance, with more female students signing up, suggesting this approach may need to be modified to engage more boys and young men who need help with these issues but are reluctant to come forward.

We found significant improvements in anxiety in the DISCOVER arm versus control. The results for reduced anxiety are consistent with those from other studies.^5^ There were also improvements in well-being, resilience and sleep, consistent with studies that have found improvements in these outcomes.^38^ This demonstrates the potential of the DISCOVER workshop to impact multiple outcomes due to its transdiagnostic approach.

### Future research

The self-referral approach can engage individuals with elevated symptoms of depression as well as those who have subthreshold depression, which is a key prognostic indicator of depression. One question is whether this approach could also act to protect subthreshold participants from transitioning to full threshold in the future; a long-term follow-up could answer this question. Further research to understand the impact of self-referral on barriers to engagement (such as stigma) could also provide valuable insights to optimise this self-referral approach.

### Strengths and limitations

The BESST study is robustly designed^21^ and was effectively conducted, reaching its recruitment target and achieving a 95% follow-up rate.^20^ However, there are limitations. Assessments were based on self-reports rather than clinical evaluations; future research should consider incorporating additional data sources, such as teacher and parent reports, as well as physiological measures. The control group was passive rather than active. Additionally, while there was a higher proportion of male students than in our feasibility study, female students remained the majority. The representativeness of the sample could be seen as a limitation as a self-referral system was used and we lack comparison data (eg, ethnicity) of the secondary school populations. Although students were followed up for 6 months, it will be important to assess if these positive outcomes remain after a longer period. Finally, given some studies have shown iatrogenic effects of mental health interventions in young people, we should note that this study did not explore these effects in any further detail than collection of adverse events and symptom scores. Future research could include assessments to identify any potential adverse effects of the intervention.

## Conclusion and clinical implications

A self-referral brief CBT intervention for 16–18-year-old adolescents in schools has been found to both reach and significantly assist with elevated depressive symptoms, both clinically and cost-effectively. This intervention has been demonstrated as an effective tool when delivered by MHSTs and could be used by the NHS as an early intervention in schools.

## Supplementary material

10.1136/bmjment-2024-301192 online supplemental file 1

## Data Availability

Data are available upon reasonable request.
