# Supplementary material for: Brief Educational Workshops in Secondary Schools Trial (BESST): a cluster randomised controlled trial. Secondary analysis in those with elevated symptoms of depression
Source: BMJ Ment Health. 2024 Aug 29;27(1):e301192. doi: 10.1136/bmjment-2024-301192 (PMC11367360; doi:10.1136/bmjment-2024-301192)

Supplementary appendix for: A cluster randomised controlled trial in Brief Educational Workshops in Secondary Schools Trial (BESST): Secondary analysis in those with elevated symptoms of depression

Appendix S1 Detailed overview of DISCOVER workshop programme	Page 2
Table S1. Baseline Clinical Characteristics	Page 4
Figure S1: Standardised effect estimates for the primary and secondary outcomes at 6 months	Page 5
Figure S2: Mean RCADS Temporal plot	Page 6
Figure S3: Mean WEMWBS Temporal plot	Page 7
Figure S4: Mean SCI Temporal plot	Page 8
Figure S5: Mean CYRM-12 Temporal plot	Page 9
Table S2. Therapy summaries	Page 10
Table S3. CSQ	Page 11
Table S4. Adverse events	Page 12
Supplementary Table S5: Disaggregated mean costs for elevated depressive symptom group (£)	Page 13
Supplementary Table S6: EQ-5D-3L scores and QALYs for elevated depressive symptom group	Page 14
Supplementary Figure S6: Scatterplot showing the bootstrapped mean differences in costs and QALYs for DISCOVER compared to control at 6-month follow-up	Page 15
Supplementary Figure S7: Cost-effectiveness acceptability curve showing the probability that DISCOVER is cost-effective compared to control in terms of QALYs at 6-month follow-up	Page 16

Appendix S1: Detailed overview of DISCOVER workshop programme

Intervention Overview
DISCOVER is a brief, accessible workshop-based stress management programme for 16–18-year-olds, to which they can self-refer. The workshop is considered accessible because of the self-referral system where students are invited to refer themselves; accessibility was also helped by using colloquial 'mental health' terms such as 'stress', and not using diagnostic terms such as 'depression' and 'anxiety'. The programme was co-designed with a Teenage Advisory Group (TAG) of 31 16-18-year-olds, with the aim of improving engagement, offering effective treatment, and maintaining participants' motivation and improvement to reduce relapse. The workshop programme includes CBT coping techniques for managing mood, anxiety, and stress, delivered in non-medicalised language, such as stress, and with images and materials including students from diverse groups.

Delivery Teams
A two-day training programme was provided for MHST staff from NHS trusts. Members of the MHSTs were trained, via day-long online sessions, to deliver the intervention in accordance with the DISCOVER manual and trial protocol. Each workshop programme was co-facilitated by three staff: one senior and two junior therapists. One supervision session between the DISCOVER trainers and each workshop delivery team was used to ensure quality and provide ongoing support. The workshop delivery teams were recruited into the trial solely for workshop delivery; they had no role in the running or design of the trial. Each MHST delivered workshops in participating schools from their NHS trust region.

DISCOVER Workshop Programme
Pre-workshop meeting:
In the days prior to attending the workshop, each student met individually with a workshop-leader in a private space at school, during school hours, for approximately 30 minutes. During this session they planned their personal goals, which they would set at the end of the workshop day.

Workshop Delivery method:
The workshop is a day-long, face-to-face group event, accommodating up to 19 students, taking place at school/college in a private classroom (without school staff present) over a single whole school day. Permission for students to attend and miss curricular activities is obtained from staff in advance, and the students' usual breaks and lunch are adhered to.

Core workshop content:
Each workshop begins with introductions and icebreakers. A CBT-informed model of emotional problems is then provided to explain and normalise young people's experiences, including video clips of teenage actors and group discussions. Particular attention is given to personal, relationship, and academic stresses typical for the age group. CBT techniques for managing anxiety and mood problems are taught and practised, supported by scripted role-plays, video demonstrations, and printed handouts. Behavioural strategies used include problem-solving, sleep advice, and time management. Cognitive strategies include identification and challenging of negative thoughts. Participants are provided with a workbook to keep, which provides all the covered material and

space to make notes throughout the workshop, as well as space to record their personal goals. They are also guided through the installation of an app, which provides this content in digital form.

Personalised follow-up:
After one week, participants are followed-up individually by one of the workshop leaders, with the participants receiving a 15-30 minute 'telephone goal reviews' to monitor progress and support incorporation of CBT skills into real-life situations. If needed, participants are given the option of receiving two further telephone goal reviews within the 12-week post-workshop period. Participants are provided with notification of the time window to expect their call via text message or email in the days prior to receiving it – they are able to reply to arrange a more suitable date/time.

Treatment Fidelity
Each member of the workshop delivery team completed a 9-item self-report fidelity checklist immediately following each workshop. An independent observer also attended one workshop per delivery team to assess treatment fidelity using the same checklist. The checklist was developed by the trial team, based on research literature and consultations with the DISCOVER workshop team. Fidelity was met if 7 out 9 items are met (including 4 mandatory items). Fidelity scores were compiled at the end trial, but not used as a means to improve fidelity throughout the trial.

Table S1. Baseline Clinical Characteristics

**Baseline Clinical Characteristics**	**Control N=172** *N (%)*	**DISCOVER N=142** *N (%)*	**Overall N= 314** *N (%)*
**Previously sought help from GP for mental health**			
No	121 (70.3)	101 (71.1)	222 (70.7)
Yes	51 (29.7)	41 (28.9)	92 (29.3)
**Had counselling or talking therapy**			
No	98 (57.0)	77 (54.2)	175 (55.7)
Yes	74 (43.0)	65 (45.8)	139 (44.3)
**If yes, was this help through school?**			
No	22 (29.7)	13 (20.0)	35 (25.2)
Yes	52 (70.3)	52 (80.0)	104 (74.8)
**If yes, was this help through CAMHS?**			
No	43 (58.1)	48 (73.8)	91 (65.5)
Yes	28 (37.8)	16 (24.6)	44 (31.7)
Missing	3 (4.1)	1 (1.5)	4 (2.9)
**Previous diagnosis of: Autism Spectrum**			
No	165 (95.9)	135 (95.1)	300 (95.5)
Yes	6 (3.5)	5 (3.5)	11 (3.5)
Missing	1 (0.6)	2 (1.4)	3 (1.0)
**ADHD**			
No	168 (97.7)	138 (97.2)	306 (97.5)
Yes	3 (1.7)	2 (1.4)	5 (1.6)
Missing	1 (0.6)	2 (1.4)	3 (1.0)
**Anxiety disorder**			
No	145 (84.3)	125 (88.0)	270 (86.0)
Yes	27 (15.7)	15 (10.6)	42 (13.4)
Missing	0 (0.0)	2 (1.4)	2 (0.6)
**Depression**			
No	156 (90.7)	124 (87.3)	280 (89.2)
Yes	16 (9.3)	16 (11.3)	32 (10.2)
Missing	0 (0.0)	2 (1.4)	2 (0.6)
**Eating disorder**			
No	164 (95.3)	130 (91.5)	294 (93.6)
Yes	7 (4.1)	8 (5.6)	15 (4.8)
Missing	1 (0.6)	4 (2.8)	5 (1.6)
**Was the study recommended by the teacher?**			
No	98 (57.0)	76 (53.5)	174 (55.4)
Yes	74 (43.0)	66 (46.5)	140 (44.6)

Figure S1. Standardised effect estimates for the primary and secondary outcomes at 6 months.

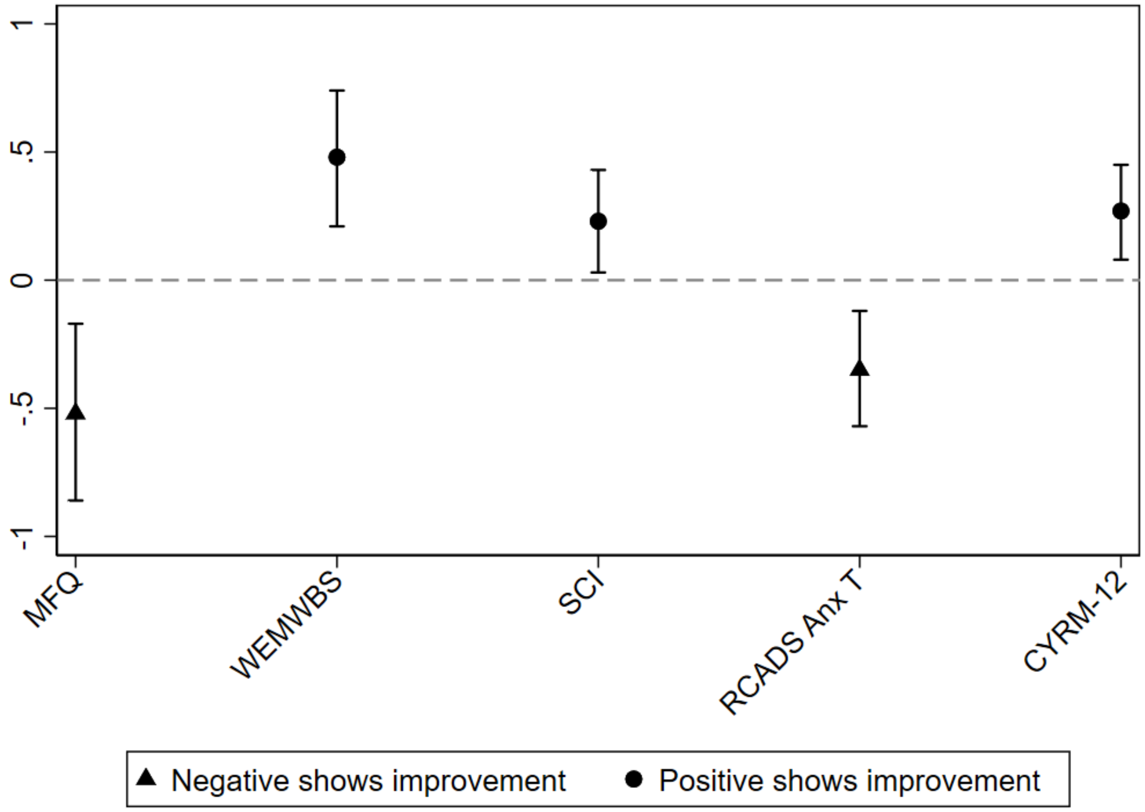

Figure S2. Mean RCADS Temporal plot

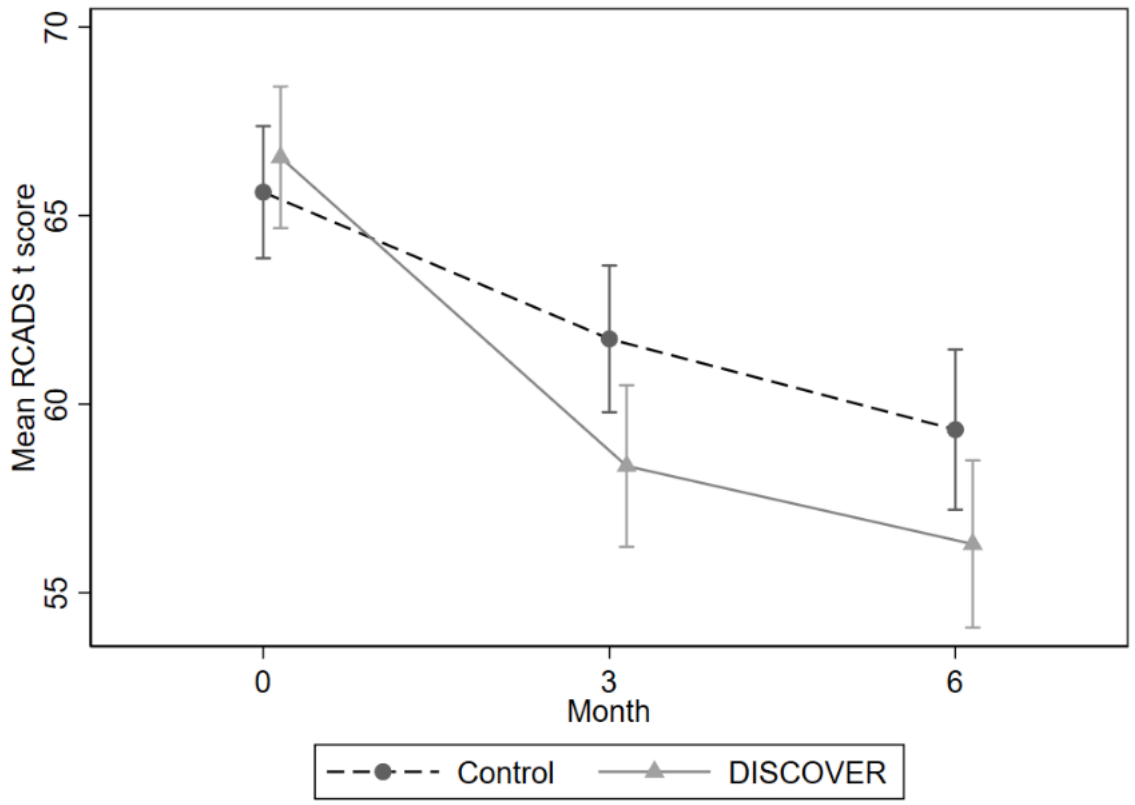

Figure S3. Mean WEMWBS Temporal plot

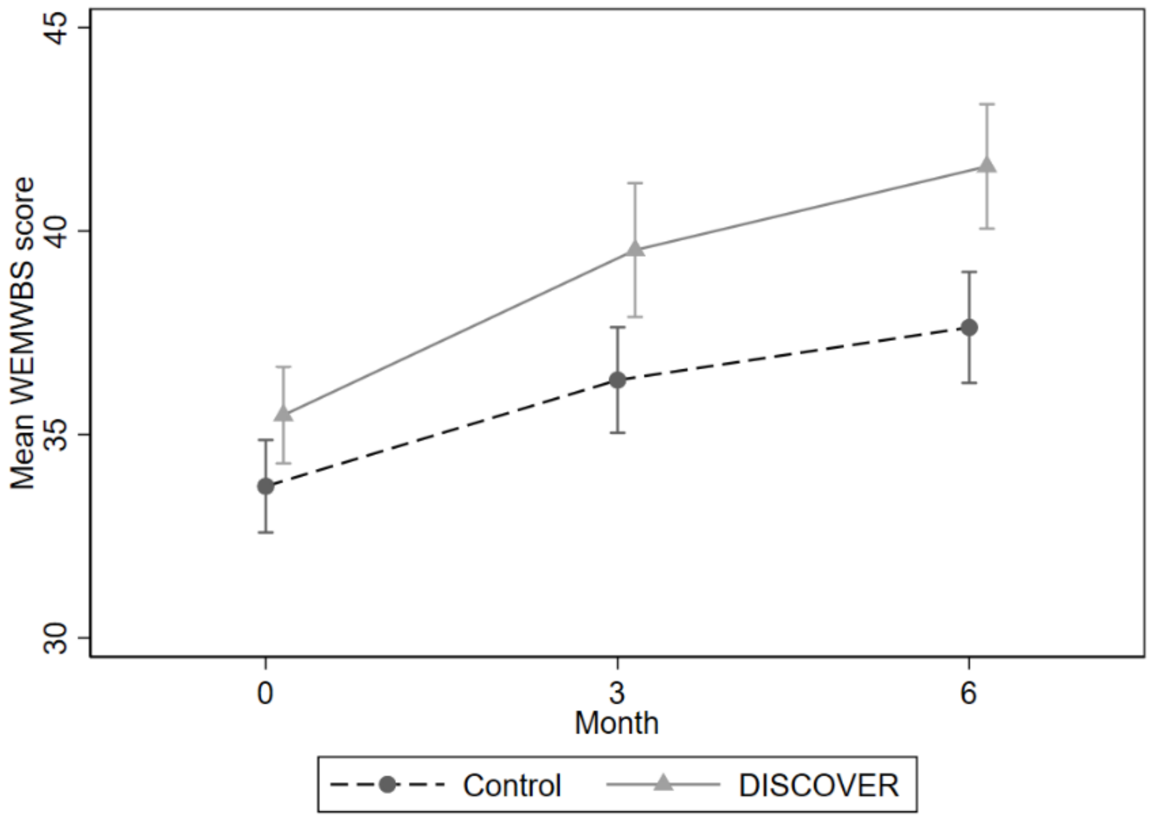

Figure S4. Mean SCI Temporal plot

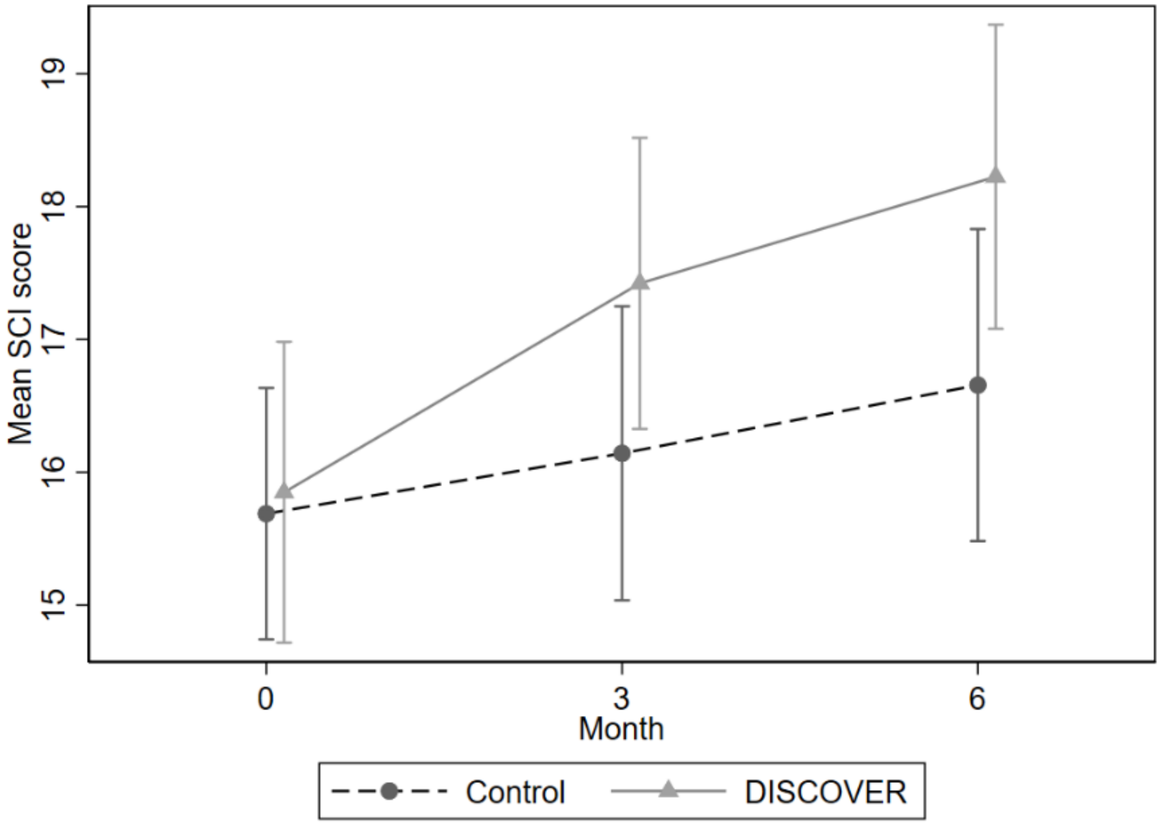

Figure S5. Mean CYRM-12Temporal plot

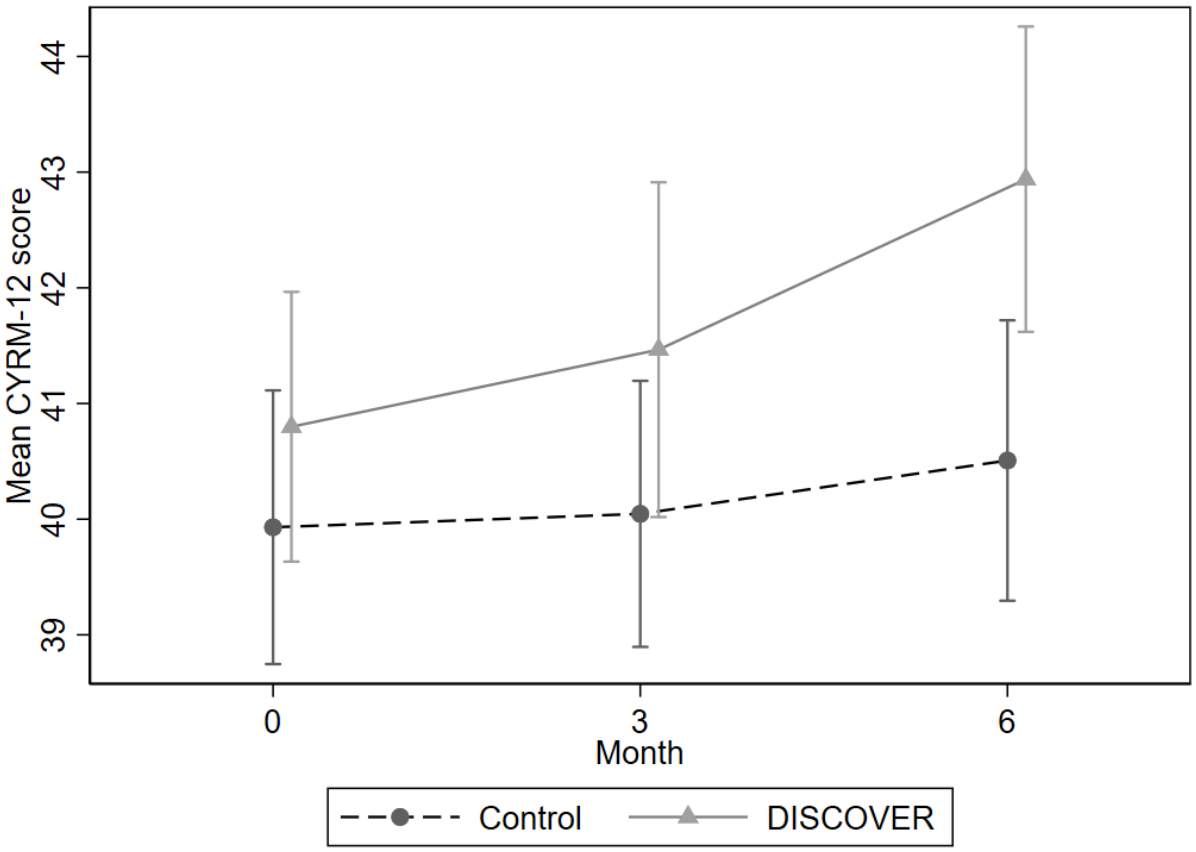

Table S2. Therapy summaries

Discover workshop information	N = 142<br>*N (%)*
**Did the participant attend pre-workshop goal planning meeting?**	
No	9 (6.3%)
Yes	133 (93.7%)
**How much of the DISCOVER workshop did the participant attend?**	
100%	114 (80.3%)
More than 75% but not the full day	9 (6.3%)
Up to 75%	3 (2.1%)
Up to 50%	2 (1.4%)
Up to 25%	1 (0.7%)
0%	13 (9.2%)
**Reason why the participant did not complete the full DISCOVER workshop day (N = 28)**	
Participant did not want to miss lessons	7 (25.0%)
Participant no longer wished to attend for another reason	2 (7.1%)
Participant did not attend school on the day of the workshop	2 (7.1%)
Participant forgot	2 (7.1%)
Other	4 (14.3%)
Missing	11 (39.3%)
**Was a goal set at the DISCOVER workshop (N=129)**	
No	3 (2.3%)
Yes	126 (97.7%)

Table S3. CSQ

**CSQ-8**	**N = 142**<br>***N (%)***
**How would you rate the quality of the service you received?**	
Poor	2 (1.4%)
Fair	4 (2.8%)
Good	47 (33.1%)
Excellent	63 (44.4%)
Missing	26 (18.3%)
**Did you get the kind of service you wanted?**	
No definitely not	2 (1.4%)
No not really	5 (3.5%)
Yes, generally	63 (44.4%)
Yes, definitely	46 (32.4%)
Missing	26 (18.3%)
**To what extent has our service met your needs?**	
None of my needs have been met	1 (0.7%)
Only a few of my needs have been met	29 (20.4%)
Most of my needs have been met	63 (44.4%)
Almost all of my needs have been met	23 (16.2%)
Missing	26 (18.3%)
**If a friend were in need of similar help, would you recommend our service?**	
No, I don't think so	9 (6.3%)
Yes, I think so	53 (37.3%)
Yes, definitely	54 (38.0%)
Missing	26 (18.3%)
**How satisfied are you with the amount of help you received?**	
Quite dissatisfied	2 (1.4%)
Indifferent or mildly dissatisfied	11 (7.7%)
Mostly satisfied	54 (38.0%)
Very satisfied	48 (33.8%)
Missing	27 (19.0%)
**Have the services you received helped you to deal more effectively with your problems?**	
No, they seemed to make things worse	7 (4.9%)
No, they really didn't help	68 (47.9%)
Yes, they helped somewhat	40 (28.2%)
Yes, they helped a great deal	27 (19.0%)
Missing	7 (4.9%)
**In an overall, general sense, how satisfied are you with the service you received?**	
Quite dissatisfied	2 (1.4%)
Indifferent or mildly dissatisfied	10 (7.0%)
Mostly satisfied	56 (39.4%)
Very satisfied	47 (33.1%)
Missing	27 (19.0%)
**If you were to seek help again, would you come back to our service?**	
No, definitely not	2 (1.4%)
No, I don't think so	16 (11.3%)
Yes, I think so	58 (40.8%)
Yes, definitely	37 (26.1%)
Missing	29 (20.4%)
**Total CSQ-8 score N = 116**	
Mean (SD)	26.1 (4.0)
Median (IQR)	27.0 (24.0-29.0)

Table S4. Adverse events

	Control<br>N = 5<br>*N (%)*	DISCOVER<br>N = 7<br>*N (%)*	Overall N = 12<br>*N (%)*
**Is the event serious**			
No	4 (80.0)	6 (85.7)	10 (83.3)
Yes	1 (20.0)	1 (14.3)	4 (16.7)
**Serious adverse event type *events(people)***			
Other psychological event (e.g., panic attack)	1 (1)	0 (0)	1 (1)
Other physiological event (e.g., injury)	0 (0)	1 (1)	1 (1)
**Adverse event type *events(people)***			
Self- harm	1 (1)	1 (1)	2 (2)
Disclosure of current abuse, physical	1 (1)	0 (0)	1 (1)
Participant became distressed	1 (1)	3 (3)	4 (4)
Other psychological event (e.g., panic attack)	2 (2)	1 (1)	3 (3)
Other physiological event (e.g., injury)	0 (0)	2 (2)	2 (2)
**Relationship to study procedures**			
Possibly related	1 (20.0)	0 (0.0)	1 (8.3)
Not related	4 (80.0)	7 (100.0)	11 (91.7)

Table S5: Disaggregated mean costs for elevated depressive symptom group (£)

	TAU<br>Mean (SD)	DISCOVER<br>Mean (SD)
**Baseline**	n=172	n=142
Hospital	106.54 (331.53)	229.23 (728.89)
Community	199.34 (497.85)	205.45 (602.54)
Medications	0.38 (3.35)	0.88 (5.65)
Total	306.26 (659.68)	435.55 (1086.06)
**Follow-up**	n=162	n=131
Hospital	465.96 (1140.09)	452.94 (1029.05)
Community	425.51 (1039.60)	640.21 (1426.17)
Medications	1.69 (10.53)	2.35 (13.12)
Total excluding DISCOVER	893.16 (1960.30)	1095.49 (2203.48)
Intervention	0.00 (0.00)	110.08 (8.69)
Total including DISCOVER	893.16 (1960.30)	1205.57 (2203.81)

Table S6: EQ-5D-3L scores and QALYs for elevated depressive symptom group

	**TAU**<br>**Mean (SD)**	**DISCOVER**<br>**Mean (SD)**
**Baseline**	n=172	n=142
EQ-5D-3L score	0.6591 (0.2319)	0.6542 (0.2429)
**Follow-up**	n=158	n=126
EQ-5D-3L score	0.6555 (0.2535)	0.7299 (0.2248)
QALYs	0.3330 (0.1070)	0.3590 (0.1029)

Figure S6: Scatterplot showing the bootstrapped mean differences in costs and QALYs for DISCOVER compared to control at 6-month follow-up

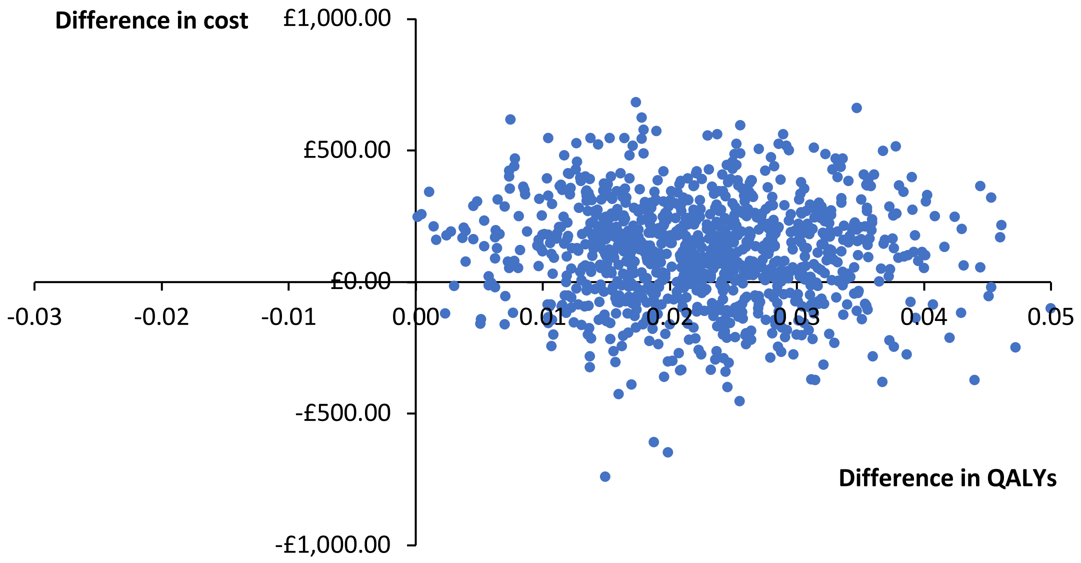

Figure S7: Cost-effectiveness acceptability curve showing the probability that DISCOVER is cost-effective compared to control in terms of QALYs at 6-month follow-up

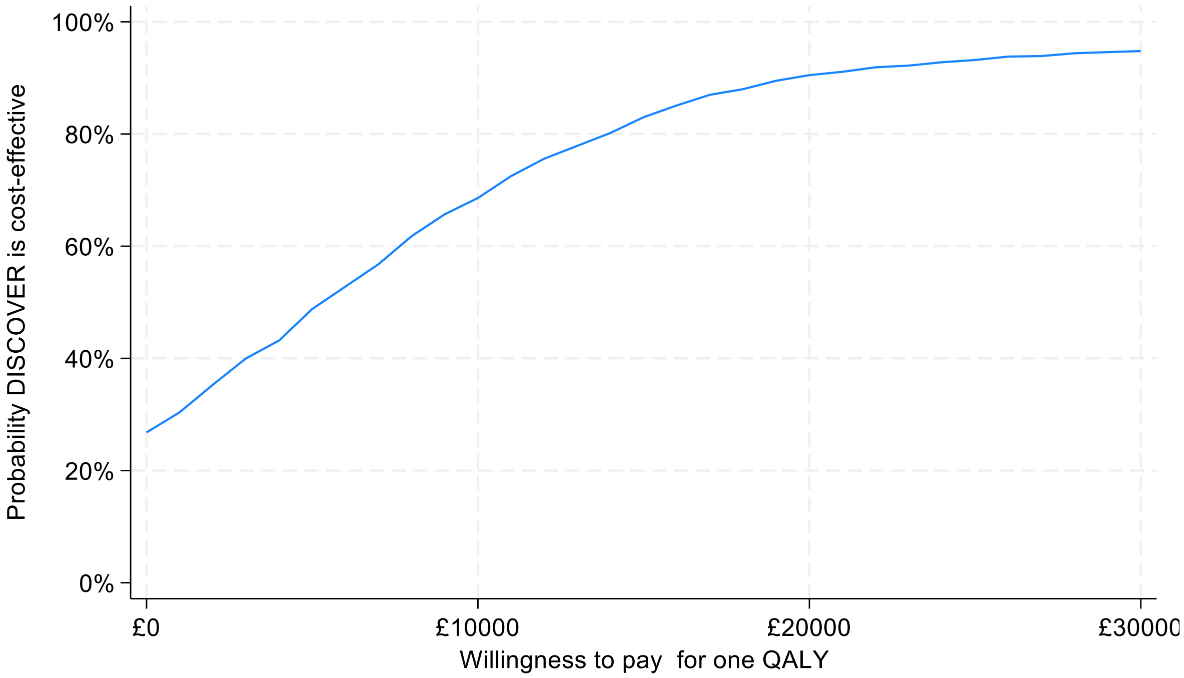

Supplement: online supplemental file 1 [file bmjment-27-1-s001.pdf]
